# Anti-TROVE2 Antibody Determined by Immune-Related Array May Serve as a Predictive Marker for Adalimumab Immunogenicity and Effectiveness in RA

**DOI:** 10.1155/2021/6656121

**Published:** 2021-03-08

**Authors:** Po-Ku Chen, Joung-Liang Lan, Yi-Ming Chen, Hsin-Hua Chen, Shih-Hsin Chang, Chia-Min Chung, Nurul H. Rutt, Ti-Myen Tan, Raja Nurashirin Raja Mamat, Nur Diana Anuar, Jonathan M. Blackburn, Der-Yuan Chen

**Affiliations:** ^1^Translational Medicine Laboratory, Rheumatic Diseases Research Center, China Medical University Hospital, Taichung, Taiwan; ^2^College of Medicine, China Medical University, Taichung, Taiwan; ^3^Rheumatology and Immunology Center, China Medical University Hospital, Taichung, Taiwan; ^4^Department of Medical Research, Taichung Veterans General Hospital, Taiwan; ^5^Faculty of Medicine, National Yang-Ming University, Taipei, Taiwan; ^6^Center for Drug Abuse and Addiction, China Medical University Hospital, Taichung, Taiwan; ^7^Graduate Institute of Biomedical Sciences, China Medical University, Taichung, Taiwan; ^8^Sengenics Corporation Pte Ltd., Singapore; ^9^Ph.D. Program in Translational Medicine, National Chung Hsing University, Taichung, Taiwan

## Abstract

Anti-drug antibody (ADAb) development is associated with secondary therapeutic failure in biologic-treated rheumatoid arthritis (RA) patients. With a treat-to-target goal, we aimed to identify biomarkers for predicting ADAb development and therapeutic response in adalimumab-treated patients. Three independent cohorts were enrolled. In Cohort-1, 24 plasma samples (6 ADAb-positive and 6 ADAb-negative patients at baseline and week 24 of adalimumab therapy, respectively) were assayed with immune-related microarray containing 1,636 correctly folded functional proteins. Next, we executed statistically powered autoantibody profiling analysis of 50 samples in Cohort-2 (24 ADAb-positive and 26 ADAb-negative patients). Subsequently, immunofluorescence assay was performed on 48 samples in Cohort-3 to correlate with ADAb titers and drug levels. The biomarkers were identified for predicting ADAb development and therapeutic response using the immune-related microarray and machine learning approach. ADAb-positive patients had lower drug levels at week 24 (median = 0.024 *μ*g/ml) compared with ADAb-negative patients (median = 6.38 *μ*g/ml, *p* < 0.001). ROC analysis based on the ADAb status revealed the top 20 autoantibodies with AUC ≥ 0.7 in differentiating both groups in Cohort-1. Analysis of Cohort-2 dataset identified a panel of 8 biomarkers (TROVE2, SSB, NDE1, ZHX2, SH3GL1, CARD9, PTPN20, and KLHL12) with 80.6% specificity, 77.4% sensitivity, and 79.0% accuracy in discriminating poor from EULAR responders. Immunofluorescence assay validated that anti-TROVE2 antibody could highly predict ADAb development and poor EULAR response (AUC 0.79 and 0.89, respectively). Multivariate regression analysis proved anti-TROVE2 antibody to be an independent predictor for developing ADAb. Immune-related protein microarray and replication analysis identified anti-TROVE2 antibody as a useful biomarker for predicting ADAb development and therapeutic response in adalimumab-treated patients.

## 1. Introduction

Rheumatoid arthritis (RA), an inflammatory articular disease, is characterized by chronic synovitis and bone erosions [1], and tumor necrosis factor- (TNF-) *α* is a crucial inflammatory mediator in this disease [2]. The important role of TNF-*α* in RA pathogenesis is supported by the effectiveness of biologics targeting this cytokine [2–4], although the efficacy diminishes in some patients over time (secondary failure) [5]. Accumulating evidence indicates that the presence of anti-drug antibodies (ADAb), the so-called immunogenicity, may be associated with low or undetectable drug levels and ensuing reduction of therapeutic responsiveness to TNF-*α* inhibitors [6–11]. Given a treat-to-target goal [12] and the uncertainty of therapeutic responsiveness to TNF-*α* inhibitors, physicians are eager to find biomarkers which can predict the emergence of ADAb and the effectiveness of biologics. However, the patient-specific predictors for biologic immunogenicity and secondary therapeutic failure have not been identified.

Proteomics research has been increasingly applied to the identification of novel biomarkers predictive of therapeutic responsiveness to biologics in RA patients [13–15]. Hagiwara et al. recently demonstrated that anti-Ro/SSA antibody-positive patients treated with infliximab (anti-TNF-*α* monoclonal antibody) had a higher proportion (50%, 3/6) of ADAb compared with anti-Ro/SSA antibody-negative patients (0%, 0/12), with all three ADAb-positive patients being poor responders to infliximab [16]. However, there is scant research into circulating potential biomarkers to predict the developing ADAb in RA patients receiving anti-TNF therapy.

The Sengenics IMMUNOME protein microarray consists of 1,636 immobilized full-length and correctly folded functional proteins that have been selected based on their involvement in the immune response [17]. This immune-related protein array enables simultaneous screening for autoantibodies against antigens and offers a powerful method to identify specific autoantibody markers [18]. This protein array has successfully identified protein biomarkers useful for predicting disease severity of dengue virus infection [19]. However, there has not been any similar study addressing the biologics immunogenicity in RA patients by using the immune-related protein array.

We aimed to identify the potential biomarkers for predicting ADAb development and therapeutic response using immune-related protein array. Subsequently, we performed a validation on the identified biomarkers in an independent cohort to test their diagnostic performance. Next, we evaluated the predictive utility of the candidate biomarkers for the emergence of ADAb and therapeutic response assessed at week 24 of adalimumab therapy in RA patients.

## 2. Materials and Methods

### 2.1. Patients and Study Design

**Figure 1:**
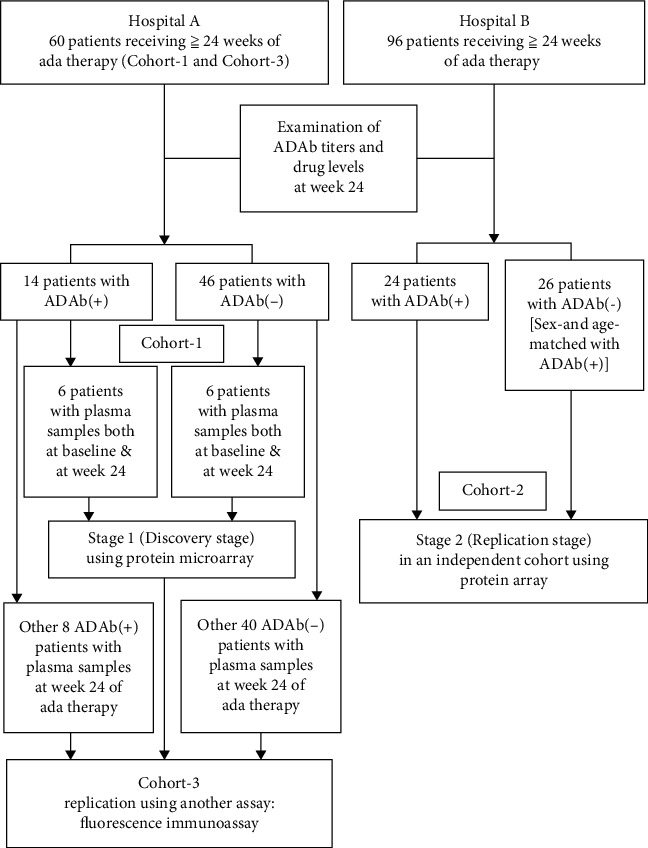
Study design and workflow. ADAb: anti-drug antibodies; ada: adalimumab.

This study is divided into two stages and three cohorts (Figure 1). In one medical center (Hospital A), 60 biologic-naïve patients who met the 2010 revised criteria of the American College of Rheumatology for RA [20] and had received at least 24 weeks of adalimumab therapy were consecutively enrolled as the combined Cohort-1 and Cohort-3. To avoid the effects of methotrexate (MTX) on the emergence of adalimumab immunogenicity [21], all patients received concomitant MTX at a stable dose of 10–15 mg weekly. Disease activity was assessed using the 28-joint disease activity score (DAS28) at baseline and week 24 of adalimumab therapy [22]. Antibodies against adalimumab (ADAb) were detected, and the therapeutic response using EULAR response criteria [23] was evaluated at week 24 of adalimumab therapy. We defined EULAR responders as patients with good and moderate EULAR therapeutic responses. The study design workflow was illustrated in Figure 1.

Stage 1 represents the pilot discovery study which is comprised of 24 plasma samples obtained from 6 ADAb(+) patients (poor responders) and 6 ADAb(−) patients (EULAR responders) at baseline and at week 24 of adalimumab therapy, respectively (Cohort-1). In stage 2, fifty independent plasma samples obtained from Cohort-2 participants including 24 ADAb(+) and 26 ADAb(−) patients selected from another medical center (Hospital B) were used to replicate findings from stage 1 and identify statistically significant biomarker panels.

We also conducted a replication study using another assay-fluorescence immunoassay. Forty-eight plasma samples from Cohort-3 (8 ADAb(+) and 40 ADAb(−) patients from Hospital A) at week 24 of adalimumab therapy were used to validate the most discriminatory biomarkers. This study was approved by the ethics committee of two medical centers (CE15285B and CMUH107-REC1-142), and the written consent was obtained from each participant according to the Declaration of Helsinki.

### 2.2. Determination of Antibodies against Adalimumab and Plasma Drug Trough Levels

Antibodies against adalimumab were detected by bridging ELISA (Progenika Biopharma, SA, Derio, Spain) at week 24 of adalimumab therapy according to the described technique [11]. This assay measures plasma levels of free ADAb but lacks sensitivity toward IgG4-ADAb because only the bivalent fraction is detected [24]. Test results were converted into arbitrary units per milliliter (AU/ml) by comparison with dilutions of a reference plasma. Patients were defined as positive for anti-adalimumab antibodies if the levels were greater than 3.5 AU/ml according to the manufacturer's instructions. To avoid the probability of a false-positive result, our patients were defined as positive if the levels were greater than the threefold value (10.5 AU/ml) of the detection limit (3.5 AU/ml). Plasma trough levels of adalimumab were determined using the sandwich ELISA (Progenika Biopharma, SA, Derio, Spain) at week 24 according to the described technique [11]. The details on these assays are added as the supplement text (available here).

### 2.3. Protein Microarray

Plasma samples were assayed using an IMMUNOME Array (Sengenics Pte Ltd., Singapore) as previously described [18, 25]. This array comprises quadruplicate spots of 1636 full-length correctly folded immobilized proteins through a proprietary biotin carboxyl carrier protein (BCCP) folding marker. Four healthy individuals were independently assayed as the normal controls. Quality control based on raw and normalized data was performed to verify the quality. This array uses Cy3-labelled biotinylated BSA (Cy3-BSA) replicates as the positive control spots across slides. The IgG dilution series also act as a positive control to assess the binding capacity of fluorescent-conjugated secondary incubation. Accurate serial dilution quantification is used as a benchmark for ensuring that labelling efficiency and spot detection pass quality control thresholds. All arrayed proteins are assayed simultaneously under identical conditions resulting in quantitative and genuinely comparative data. Raw data are processed and normalized using a robust pipeline that has been previously described [26]. In the data sheet generated from array, both foreground and background intensities of each spot are represented in relative fluorescence units (RFUs). Individual fold change is calculated by dividing the RFU value for each protein by the mean RFU value of each protein. We performed the composite normalization of data using both quantile-based and intensity-based normalization based on the Cy3-BSA control spots [27].

### 2.4. Identification of Discriminative Biomarkers for the Presence or Absence of ADAb

In protein microarray, the intensity of each spot was represented in relative fluorescent units (RFUs). The autoantibody response towards antigens on the array is classified as a biomarker if it satisfied all of the following criteria: (i) high penetrance fold change in the tested group (pFC ≥ 2.0-fold), (ii) high penetrance frequency in the tested group (pFreq ≥ 20%), and (iii) low penetrance frequency % in the pooled negative control group (pFreq < 10%). The top 20 potential biomarkers at baseline and at week 24 of adalimumab therapy, which could stratify between ADAb-positive and ADAb-negative patients, were selected based on penetrance fold change. To test the diagnostic ability, individual receiver operating characteristic (ROC) curve analysis for the differentially expressed markers was performed to determine the area under ROC curve (AUC). The high discriminative biomarkers were selected from the overlapped markers with AUC≧0.80 in the ROC analysis at both time points. Using the composite-normalized RFU of all 1,622 antigens, a ROC analysis was performed to determine the sensitivity and specificity of each antigen in differentiating both groups. The ROC statistics are calculated based on “roc” function from the “pROC” package in R. The unsupervised clustering analysis to generate the heat map was conducted using Ward's method, and the distance was calculated based on the Euclidean distance.

### 2.5. Biomarker Panel Selection and Bioinformatics

The extracted raw data from Cohort-2 was merged with those from Cohort-1 baseline samples, and ComBat normalization was performed on the net fluorescent intensity values to adjust for possible batch effects between the two studies [28]. The ComBat-normalized fluorescent intensities across 1,600+ autoantibodies were used as inputs for model generation. The best subsets of biomarkers were selected based on a backward selection iterative process known as recursive feature elimination (RFE). The selection is classified based on the ADAb status. Permutating features (autoantibodies) with high mean decrease in Gini importance would result in reduced separation of the two output classes. The 300 iterations were performed with random resampling and fivefold crossvalidation on the dataset. This methodology was adapted as an exhaustive way to identify the best panel of autoantibodies while considering randomization in the dataset. Using a machine learning approach, RA cohorts were randomly divided into a training dataset and a test dataset and a confusion matrix was built using the selected variables to summarize the performance of the classification algorithm.

To test the stability of the best panel selected from RFE, random forest (RF) analysis was performed with the number of trees generated being set to 1,000 and the number of features being set to the default value [29, 30]. One thousand iterations of RF were performed to assess the overall stability of the model.

### 2.6. Statistical Analyses

Fisher's exact test was used for between-group comparisons of ADAb positivity and therapeutic responses. The Mann–Whitney *U* test was used for among-group comparison of drug levels or plasma levels of candidate biomarkers between ADAb-positive and ADAb-negative patients. Spearman's correlations were determined between plasma levels of candidate biomarkers and ADAb titers. We constructed a multivariate logistic regression model to evaluate the factors predicting the emergence of immunogenicity or a poor EULAR response. The ROC curve analysis was performed to determine the area under ROC curve (AUC), sensitivity, specificity, and accuracy using MedCalc v.14. A *p* value < 0.05 was considered significant.

## 3. Results

### 3.1. Clinical Characteristics of RA Patients

Among the 60 patients in the combined Cohort-1 and Cohort-3, 14 (23.3%) patients had positive ADAb assessed at week 24 of adalimumab therapy. The positive rate of anti-Ro60/SSA antibody at baseline was significantly higher in ADAb-positive patients (64.3%) than in ADAb-negative patients (4.3%, *p* < 0.001). However, there were no significant differences in the positive rate of rheumatoid factor or anti-citrullinated peptide antibodies, dosage of corticosteroids or MTX, or the proportion of csDMARD usage between the ADAb-positive and ADAb-negative patients (Table 1).

In an independent Cohort-2 of 50 samples (24 ADAb-positive and 26 ADAb-negative patients), the differences in clinical characteristics between ADAb-positive and ADAb-negative groups (Supplementary Table S1) were similar to those in the aforementioned combined cohort.

### 3.2. ADAb Titers and Their Relation to Adalimumab Levels or Therapeutic Response

**Figure 2:**
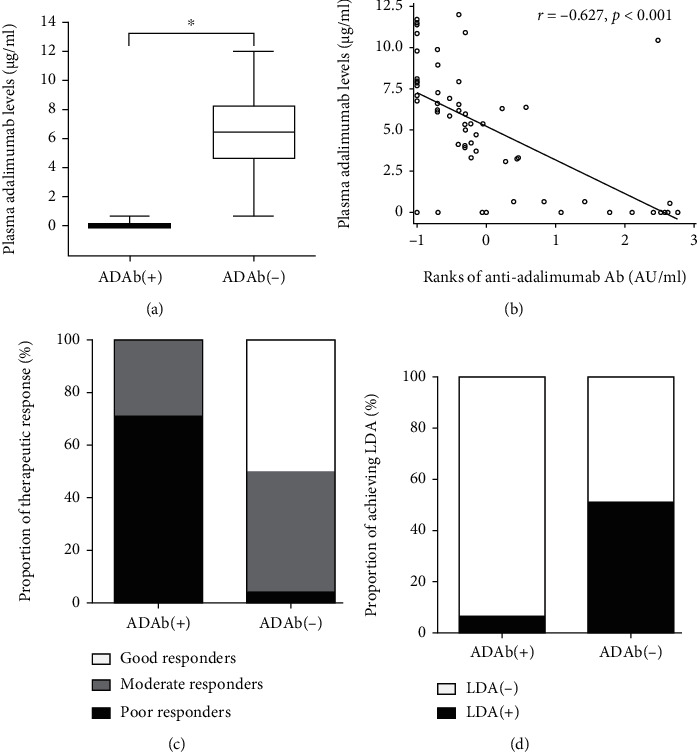
Comparison of plasma drug levels between RA patients with positive ADAb [ADAb(+)] and negative ADAb [ADAB(−)] assessed at week 24 of adalimumab therapy (a). The correlation between plasma ADAb titer and drug levels (b). The proportion of EULAR therapeutic response (c) and the proportion of achieving low disease activity (d) at week 24 in RA patients with ADAb(+) and ADAb(−). The ranks of the anti-adalimumab Ab value were presented as the log transformation. RA: rheumatoid arthritis; EULAR: European League Against Rheumatism. Good EULAR responders are defined as patients who have a decrease in DAS28 from baseline (*∆*DAS28) > 1.2 and a DAS28≦3.2; moderate responders have either *∆*DAS28 > 1.2 and a DAS28 > 3.2 or *∆*DAS28 of 0.6-1.2 and a DAS28≦5.1; poor responders are those who have either *∆*DAS28 < 0.6 or a DAS28 > 5.1. Low disease activity was defined as DAS28≦3.2. The data were presented as boxplot diagrams, with the box encompassing the 25^th^ percentile (lower bar) to the 75^th^ percentile (upper bar). The horizontal line within the box indicates the median value and the horizontal lines above and below the box represent the maximum and minimum values, respectively, for each group. ^∗^*p* < 0.001, ADAb(+) versus ADAb(−) patients.

As shown in Table 1 and Figure 2(a), ADAb-positive patients had significantly lower drug levels at week 24 of adalimumab therapy compared with ADAb-negative patients. There was also an inverse correlation between ADAb titers and drug levels (Figure 2(b)). Hence, ADAb-positive patients had a significantly higher proportion of poor EULAR response and lower proportion of achieving low disease activity at week 24 compared with ADAb-negative patients (Table 1 and Figures 2(c) and 2(d)).

### 3.3. Individual Biomarker Analysis Identified That TROVE2 Is Highly Associated with Poor Therapeutic Response

**Figure 3:**
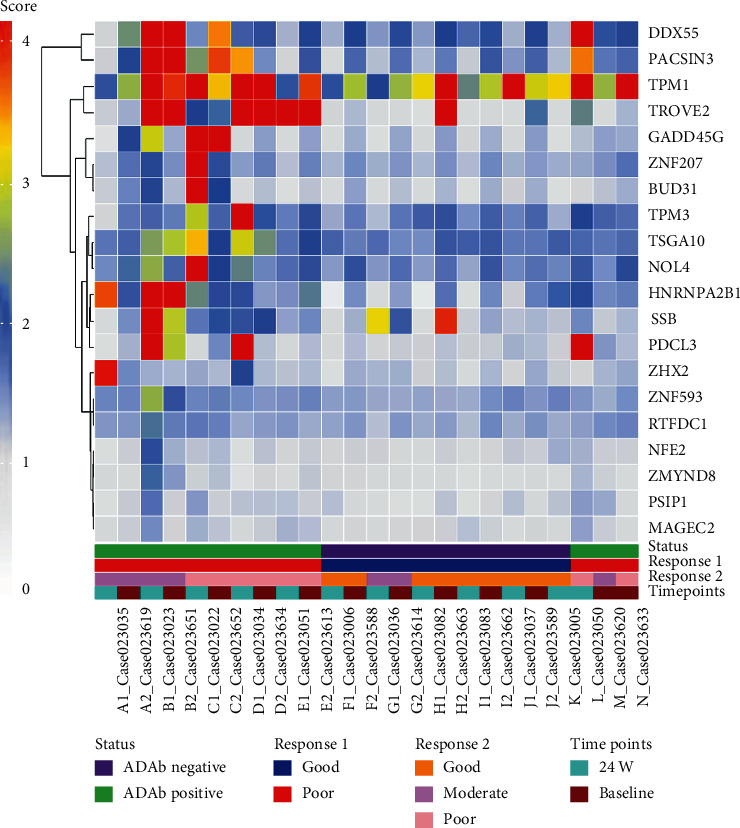
Heat map protein expression and hierarchical clustering of the top 20 putative markers for the presence of anti-drug antibody (ADAb) or poor EULAR response in 24 plasma samples from 12 patients at baseline and week 24 of adalimumab therapy, respectively. The expression level of the differentially expressed proteins was presented as the relative fluorescence unit (RFU) plotted with different colors.

Based on penetrance fold change analysis on the pilot study data, the top 20 putative biomarkers at baseline and at week 24 that are able to stratify between ADAb-positive and ADAb-negative patients are listed in Supplementary Table S2. Nine of these biomarkers (TROVE2, PACSIN3, SSB, DDX55, HNRNPA2B1, GADD45G, PDCL3, TSGA10, and TPM1) overlapped between baseline and week 24 (Figure 3).

A multiple logistic regression was calculated to predict the presence of ADAb based on 20 biomarkers (BUD31, DDX55, GADD45, HNRNPA2B1, MAGEC2, NFE2, NOL4, PACSIN3, PDCL3, PSIP1, RTFDC1, SSB, TPM1, TPM3, TROVE2, TSGA10, ZHX2, ZMYND8, ZNF207, and ZNF593). Fifteen out of 20 biomarkers were considered as significant, and anti-TROVE2 antibody had the highest discriminative power as shown in Supplementary Table S3.

**Figure 4:**
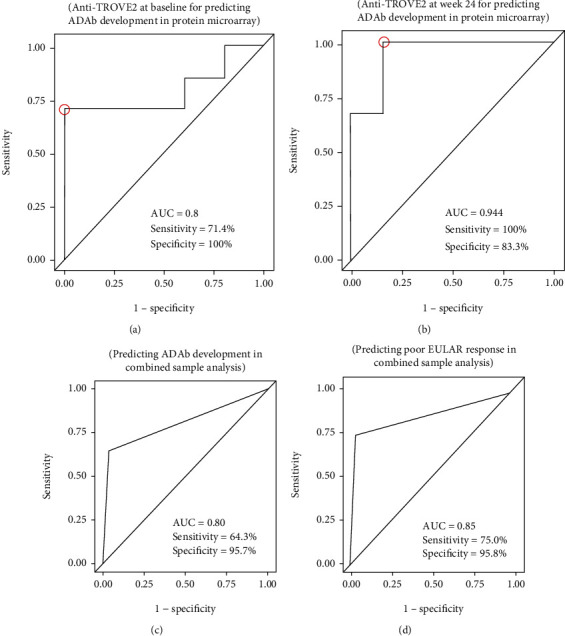
Receiver operating characteristic (ROC) curve analysis of the candidate biomarker, anti-TROVE2 (Ro60/SSA) antibody, either (a) at baseline or (b) at week 24 for predicting the emergence of anti-drug antibody (ADAb) in protein microarray. ROC curve analysis of anti-Ro60/SSA antibody at baseline for predicting the emergence of (c) ADAb or (d) poor EULAR response in combined sample analysis. AUC: area under ROC curve. Poor EULAR responders are defined as patients who have either *∆*DAS28 < 0.6 or a DAS28 > 5.1 assessed at week 24 of adalimumab therapy.

Individual ROC analysis of the putative biomarkers revealed that anti-TROVE2 antibody had a high discriminative power both at baseline and at week 24 for predicting the emergence of ADAb (Figures 4(a) and 4(b)). In combined sample (Cohort-1 and Cohort-3) analysis, anti-TROVE2 antibody at baseline also had a high discriminative power for predicting the emergence of ADAb and poor EULAR response (Figures 4(c) and 4(d)).

### 3.4. Plasma TROVE2 Antibody Titers and Their Relation to ADAb Titers or Drug Levels

**Figure 5:**
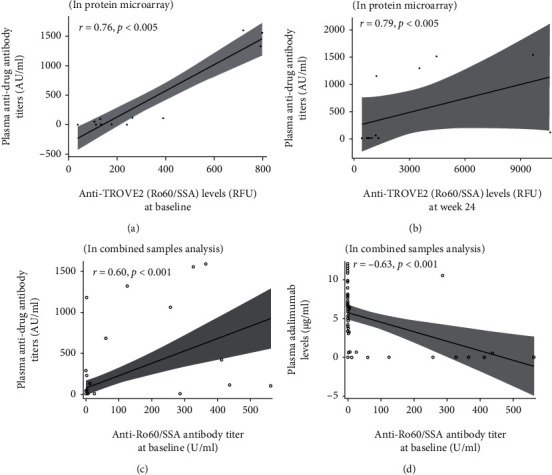
The correlation between anti-TROVE2 (Ro60/SSA) antibody titers either at baseline (a) or at week 24 (b) and plasma anti-drug antibody (ADAb) titers in the protein microarray. The correlation between anti-Ro60/SSA antibody titers at baseline and plasma anti-drug antibody (ADAb) titers (c) or plasma drug levels in combined samples analysis (d). Correlation coefficient (*R*) was calculated by the Spearman rank correlation test.

There was a positive correlation between anti-TROVE2 antibody titers either at baseline or at week 24 and ADAb titers (*r* = 0.76, *p* < 0.005 and *r* = 0.79, *p* < 0.005, respectively (Figures 5(a) and 5(b))). In combined samples, anti-TROVE2 antibody titers at baseline were positively correlated with ADAb titers (*r* = 0.60, *p* < 0.001) and negatively correlated with plasma adalimumab levels (*r* = −0.63, *p* < 0.001 (Figures 5(c) and 5(d))).

### 3.5. Plasma TROVE2 Antibody Titers and Their Relation to EULAR Response

In combined sample analysis, plasma anti-TROVE2 antibody titers were significantly higher in poor EULAR responders (median = 125.0 U/ml, IQR = 12.0–365.0 U/ml) than in EULAR responders (median = 0.40 U/ml, IQR = 0.10–0.70 U/ml, *p* < 0.001). There was an inverse correlation between anti-TROVE2 antibody titers at baseline and *∆*DAS28 assessed at week 24 in adalimumab-treated patients (*r* = −0.501, *p* < 0.001).

### 3.6. Multivariate Logistic Regression for Predicting ADAb Development or Therapeutic Response

Among 60 RA patients from the combined Cohort-1 and Cohort-3, multivariate logistic regression analysis identified anti-TROVE2 antibody to be an independent biomarker associated with ADAb development (odds ratio (OR) 70.27, 95% confidence interval (CI) 8.17-604.38, *p* < 0.001) after adjusting for age, sex, disease duration, radiographic stage, baseline DAS28, and the positivity of RF or ACPA. Similarly, anti-TROVE2 antibody was found to be an independent biomarker for predicting poor EULAR response assessed at week 24 (OR 55.1, 95% CI 1.9-1596.3, *p* < 0.05) after adjusting for potential confounding factors.

### 3.7. Recursive Feature Elimination (RFE) to Identify the Panel of Biomarkers with the Highest Discriminative Power

In order to identify additional biomarkers able to predict the development of ADAbs, we carried out a larger, statistically powered study on additional plasma samples (Cohort-2) using the same IMMUNOME Array platform. The resultant dataset was merged with the pilot baseline dataset in order to further increase statistical power in the subsequent machine learning RFE and RF analyses. Following ComBat normalization to reduce batch effects, normalized net intensity values across all proteins on the IMMUNOME Array were subjected to RFE to identify and rank the individual biomarkers based on their ability to discriminate. The importance of each biomarker is shown in Supplementary Figure 1, with the most important variables, TPBG, TROVE2, and SSB, being indicated at the left hand end of the plot.

In addition, RFE analysis was conducted to identify the best panels of biomarkers based upon their performance in stratifying the samples according to the ADAb status. Supplementary Table S4 shows the 7 top panels identified in the larger cohort study. Notably, all 7 panels contain at least TROVE2 and SSB.

**Figure 6:**
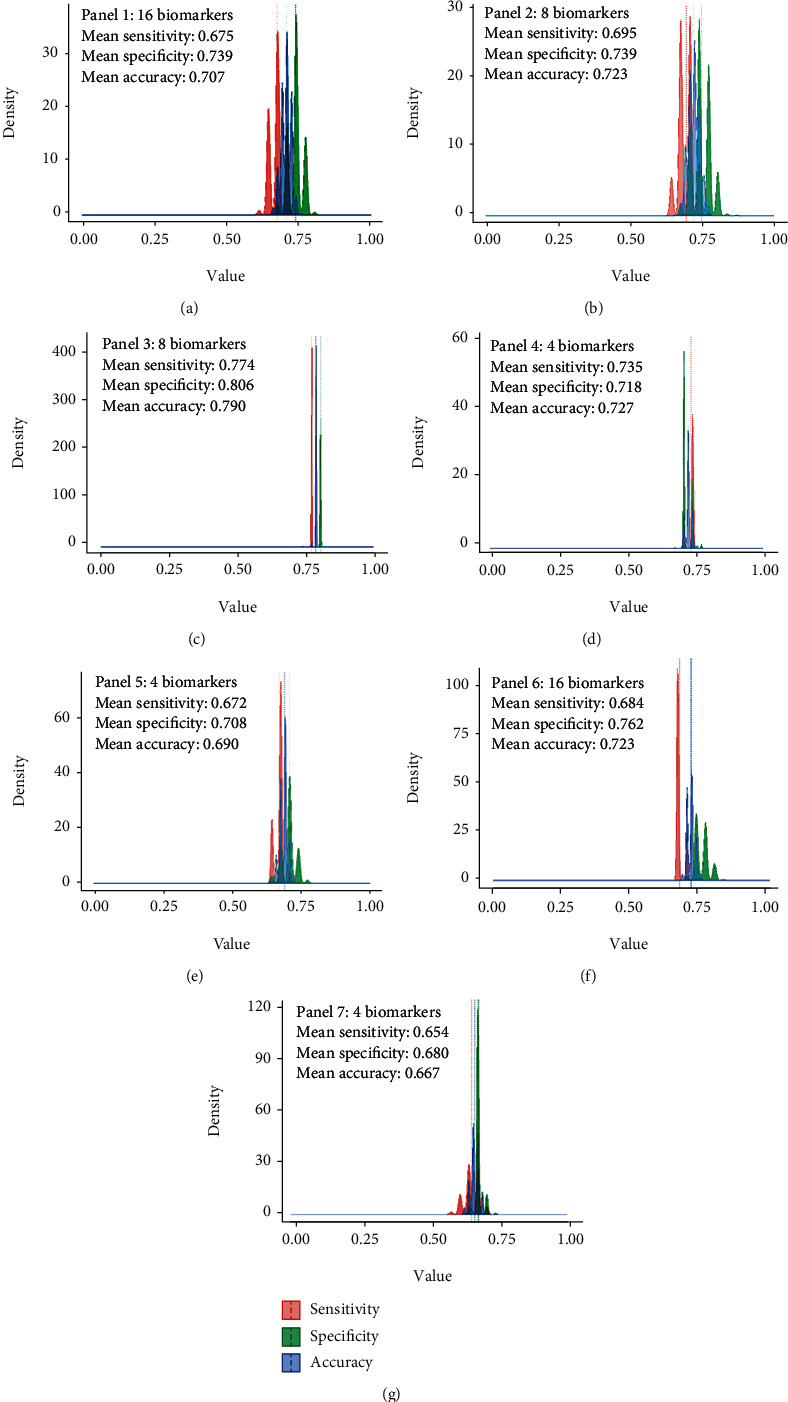
Mean distribution plot for 1,000 iterations from random forest analysis. (a)-(g) The most stable panel with 8 biomarkers which consists of TROVE2, SSB, NDE1, ZHX2, SH3GL1, CARD9, PTPN20, and KLHL12.

To determine the stability of the 7 panels, random forest analysis was performed based on the normalized net intensity values of the best predictors and subjected into 1000 iterations. Randomization of the samples is based on the feature reduction classification method using an RF algorithm. Analysis showed that one panel consisting of 8 biomarkers (TROVE2, SSB, NDE1, ZHX2, SH3GL1, CARD9, PTON20, and KLHL12) gave the most stable performance, with 80.6% specificity, 77.4% sensitivity, and 79.0% accuracy (Figure 6(c)). Although TROVE2 and SSB were found in all panels, the diagnostic performance of this panel (Supplementary Figure 2) was found to be lower than that in the panel 3 of 8 biomarkers.

### 3.8. Change in Plasma Anti-TROVE2 Levels after 6-Month Adalimumab Therapy

The change in anti-TROVE2 levels determined by fluorescence immunoassay after 6-month therapy was analyzed, and no statistical significance was found (mean ± SD, 79.0 ± 28.2 U/ml and 97.0 ± 30.1 U/ml; Supplementary Figure 3).

## 4. Discussion

Currently, there are no definite circulating protein biomarkers that can reliably predict the emergence of ADAb or therapeutic responsiveness to adalimumab. The immune-related protein microarray platform used in this study offers a powerful tool to identify autoantibody-based diagnostic or prognostic biomarkers in many diseases and treatments; particularly, it displays both linear and discontinuous epitopes across thousands of human proteins, thus enabling the identification of specific autoantibody markers. Using this platform, we are the first to identify a novel panel of 8 autoantibodies able to discriminate between patients with and without ADAb with high sensitivity (77%) and specificity (81%). Amongst these autoantibodies, anti-TROVE2 antibody had the highest individual discriminating ability, both at baseline (pretreatment) and week 24 of adalimumab therapy, and may serve as a novel predictor of adalimumab immunogenicity. In the independent validation cohort, the use of immunofluorescent-based ELISA confirmed that anti-TROVE2 performed particularly well in discriminating ADAb-positive patients from ADAb-negative patients. Moreover, plasma anti-TROVE2 antibody levels were correlated positively with ADAb titers and negatively with drug levels. Besides, the multivariate regression analysis revealed that anti-TROVE2 antibody was an independent predictor for ADAb development and poor EULAR response.

Given the association of ADAb positivity with reduced therapeutic response, it is not surprising that a higher proportion of our RA patients with anti-TROVE2 (anti-Ro60/SSA) antibody had poor EULAR response compared to those without this antibody. Other previous studies [16, 31] have reported a significant association of anti-Ro60/SSA positivity with treatment failure to infliximab, a biologic with high immunogenicity [32, 33] like adalimumab. The strong association of anti-TROVE2 with developing ADAb to adalimumab in our study may also explain the lack of significant association of anti-Ro60/SSA positivity with therapeutic inefficiency of etanercept, abatacept, or tocilizumab [16, 31], which have low immunogenicity [11, 32–35]. Based on these findings, we propose that the detection of anti-TROVE2 (anti-Ro60/SSA) antibody before starting therapy with biologics, especially those with high immunogenicity, may predict a high rate of secondary therapeutic failure due to the development of ADAb.

Autoantibodies against the 60 kDa Ro (Ro60)/SSA ribonucleoprotein (TROVE2), which is a common member of extractable nuclear antigens and frequent target of humoral immunity, are usually detected in autoimmune diseases such as primary Sjogren's syndrome, systemic lupus erythematosus, and RA. Anti-Ro60/SSA antibodies were found in 3–16.8% of RA patients [31, 36–38], a rate similarly observed in our study (18.3%). Given the close link between anti-TROVE2 antibody and the development of ADAb in our adalimumab-treated patients, we speculate that Ro autoantigen probably plays a role in the diversification of the autoantibody response through a determinant spreading [39]. Similarly, Deshmukh et al. identified multiple T cell and B cell determinants contained in both human and mouse Ro60 peptides, which can enhance the autoimmune responses of T cells and B cells [40] and may support our hypothesis. Mamula et al. revealed that Ro/SSA and the F(ab′) fragment of immunoglobulin G shared epitopes that were bound by anti-Ro/SSA antibody [41]. Moreover, it has been reported that anti-Ro/SSA antibody-positive RA patients had B cell activation with a spread autoantibody profile, including polyclonal hypergammaglobulinemia and positive antinuclear antibodies [38, 42, 43]. Magill et al. recently reveal that a low percentage of signal regulatory protein *α*/*β* + memory B cells in peripheral blood can predict the development of ADAb to adalimumab [44]; however, the mechanism remains unclear. These findings suggest that anti-Ro60 (anti-TROVE2) autoantibody directly binds to the fully human monoclonal antibody adalimumab, although the correlation coefficient between anti-Ro60 titers and ADAb titers might be expected to be higher than 0.79 measured in our study. Presumably, an initially weak binding interaction between anti-Ro60 (anti-TROVE2) and adalimumab could potentially serve as a template for ensuing development of anti-drug antibodies with high affinity adaptive response. However, an exact mechanism of anti-TROVE2 in the development of neutralizing ADAb responses remains to be explored.

Despite the novel findings presented in this pilot study, there are some limitations. Given a limited data for ADAb-positive samples, the prediction scores were built on training and testing datasets randomly split from our cohort dataset; however, we revealed a good replication of the prediction scores in an independent cohort. Therefore, a future study enrolling a larger group of ADAb-positive RA patients is needed.

## 5. Conclusion

Recently, an integrated analysis of blood-based biomarkers with clinical data requires an adaptation of the machine leaning approach [45]. We are the first to use high-density protein microarray with the machine learning approach to identify protein biomarkers that are predictive of ADAb development and therapeutic response to adalimumab. Our findings indicate that anti-TROVE2 (anti-Ro60/SSA) antibody could be a useful baseline biomarker for predicting the emergence of ADAb and secondary therapeutic failure in adalimumab-treated RA patients. Our findings provide aid in making personalized therapeutic decisions with cost-effective benefit.

## Figures and Tables

**Table 1:** Clinical characteristics of RA patients with and without ADAb in the combined Cohort-1 and Cohort-3 (Hospital A)^a^.

	ADAb positive (*n* = 14)	ADAb negative (*n* = 46)	*p* value
Mean age at entry of study (years)	55.2 ± 13.4	56.7 ± 13.5	0.728
The proportion of female (%)	13 (92.9%)	39 (84.8%)	0.667
Disease duration (years)	13.0 ± 5.3	12.4 ± 5.2	0.638
RF positivity (%) at baseline	11 (78.6%)	35 (76.1%)	0.580
ACPA positivity (%) at baseline	10 (71.4%)	31 (67.4%)	0.526
Anti-Ro60/SSA positivity (%) at baseline	9 (64.3%)	2 (4.3%)	<0.001
DAS-28 at baseline	6.71 ± 0.79	6.45 ± 0.75	0.274
Daily steroid dose (mg) at baseline	7.1 ± 2.2	6.8 ± 1.7	0.596
Weekly MTX dose (mg) at baseline	12.9 ± 2.4	13.0 ± 2.2	0.848
csDMARDs at baseline			
Methotrexate	14 (100%)	46 (100%)	—
Sulfasalazine	11 (80.0%)	38 (82.6%)	0.707
Hydroxychloroquine	10 (71.4%)	35 (76.1%)	0.734
Plasma ada levels at week 24 (*μ*g/ml)	0.02 (0.02–0.02)	6.38 (4.59–8.31)	<0.001
Poor EULAR responder at week 24	10 (71.4%)	2 (4.3%)	<0.001
The proportion of LDA at week 24	2 (14.3%)	23 (50.0%)	0.028

^a^Data are presented as mean ± standard deviation, number (percentage), or median (interquartile range). RF: rheumatoid factor; ACPA: anti-citrullinated peptide antibodies; DAS28: disease activity score for 28 joints; MTX: methotrexate; DMARDs: disease-modifying antirheumatic drugs; ada: adalimumab; LDA: low disease activity, which was defined as DAS28≦3.2; EULAR: European League Against Rheumatism. Poor responders are those who have either *∆*DAS28 (DAS28 decrement) < 0.6 or a DAS28 > 5.1 at week 24 of adalimumab therapy. Mann–Whitney *U* test was used for between-group comparison of numerical variables. The *χ*^2^ test with Yates's continuity correction or Fisher's exact test was used to compare binary variables.

## Data Availability

All data relevant to the study are included in the article or uploaded as supplementary materials.

## Abbreviations

ADAb: Anti-drug antibodies
AUC: Area under ROC curve
ada: Adalimumab
ACPA: Anti-citrullinated peptide antibodies
BCCP: Biotin carboxyl carrier protein
DAS28: Disease activity score for 28 joints
DMARDs: Disease-modifying antirheumatic drugs
EULAR: European League Against Rheumatism
LDA: Low disease activity
MTX: Methotrexate
RFE: Recursive feature elimination
RFUs: Relative fluorescence units
RF: Rheumatoid factor
RF: Random forest
TNF-*α*: Tumor necrosis factor-*α*.

## Supplementary Materials

Supplementary Materials
*Assessments of plasma levels of antibodies against adalimumab.* Antibodies against adalimumab were detected by bridging ELISA (Progenika Biopharma, SA, Derio, Spain). Briefly, 100 *μ*l of samples was added to ELISA-corresponding wells precoated with adalimumab and then incubated for 1 hour at room temperature (RT) (20–25°C). After removal of the content of each well by decanting, 200 *μ*l of diluted wash buffer was added and later removed by decanting for a total of 3 washes. Subsequently, 100 *μ*l of horseradish peroxidase- (HRP-) conjugated adalimumab was added to each well and then incubated for 1 hour at RT. After 3 washes with diluted wash buffer solution, 50 *μ*l of substrate solution was added to each well and incubated for 30 minutes at RT. Finally, 50 *μ*l of stop solution was added to each well and then thoroughly mixed. The absorbance (OD) to a 450 nm wavelength in each well was read within 15 minutes. Patients were defined as positive for anti-adalimumab antibodies if the levels were greater than the threefold value (10.5 AU/ml) of the detection limit (3.5 AU/ml) on at least 1 occasion. The overall intra-assay and interassay coefficients of variation (CV) were calculated at 6.6% and 11.8%, respectively. All baseline samples taken before starting adalimumab therapy were negative for anti-adalimumab antibody. *Determination of plasma trough levels of adalimumab.* Plasma trough levels of adalimumab were determined using sandwich ELISA according to the manufacturer's instructions (Progenika Biopharma, SA, Derio, Spain). A human monoclonal F(ab′)_2_ fragment to adalimumab was engineered in this assay, in contrast to polyclonal antibodies used in other assays. In brief, 100 *μ*l of plasma samples was added to the wells precoated with an anti-adalimumab human F(ab′)_2_ fragment and then incubated for 1 hour at RT. After removal of the content of each well by decanting, 200 *μ*l of diluted wash buffer was added and later removed by decanting for a total of 3 washes. After elimination of the diluted wash buffer solution, 100 *μ*l of HRP-labeled anti-adalimumab F(ab′)_2_ fragment was added to each well and incubated for 1 hour at RT. After 3 washes with diluted wash buffer solution, 50 *μ*l of substrate solution was added to each well and then incubated in the dark for 30 minutes at RT. Finally, 50 *μ*l of stop solution was added to each well and thoroughly mixed. The absorbance (OD) to a 450 nm wavelength in each well was read within 15 minutes. The minimal detectable levels were 0.024 *μ*g/ml for adalimumab. The overall intra-assay and interassay CV were calculated at 6.1% and 5.1%, respectively. Supplementary Table S1: clinical characteristics of RA patients with and without ADAb in Cohort-2 (Hospital B)^a^. Supplementary Table S2: the top 20 putative biomarkers which can stratify RA patients based on the ADAb status. Supplementary Table S3: a multiple logistic regression was calculated to predict the presence of ADAb based on the 20 biomarkers. Supplementary Table S4: the panels of biomarkers in stratifying anti-drug antibody- (ADAb-) positive and ADAb-negative RA patients. Supplementary Figure S1: the ranking of the discriminative power for the anti-drug antibody (ADAb) status among the individual biomarkers based on the mean decrease in Gini value. Supplementary Figure S2: mean distribution plot and the stability of a panel of both TROVE2 and SSB. Supplementary Figure 3: change in plasma anti-TROVE2 levels after 6-month adalimumab therapy. The change in anti-TROVE2 levels determined by fluorescence immunoassay before and after 6-month therapy was analyzed, and no statistical significance was found. Rx: treatment. Bars and error bars indicate mean value and standard deviation, respectively.
