# Supplementary material for: Anti-TROVE2 Antibody Determined by Immune-Related Array May Serve as a Predictive Marker for Adalimumab Immunogenicity and Effectiveness in RA
Source: J Immunol Res. 2021 Mar 8;2021:6656121. doi: 10.1155/2021/6656121 (PMC7963899; doi:10.1155/2021/6656121)
Supplement: Supplementary Materials — Assessments of plasma levels of antibodies against adalimumab. Antibodies against adalimumab were detected by bridging ELISA (Progenika Biopharma, SA, Derio, Spain). Briefly, 100 μl of samples was added to ELISA-corresponding wells precoated with adalimumab and then incubated for 1 hour at room temperature (RT) (20–25°C). After removal of the content of each well by decanting, 200 μl of diluted wash buffer was added and later removed by decanting for a total of 3 washes. Subsequently, 100 μl of horseradish peroxidase- (HRP-) conjugated adalimumab was added to each well and then incubated for 1 hour at RT. After 3 washes with diluted wash buffer solution, 50 μl of substrate solution was added to each well and incubated for 30 minutes at RT. Finally, 50 μl of stop solution was added to each well and then thoroughly mixed. The absorbance (OD) to a 450 nm wavelength in each well was read within 15 minutes. Patients were defined as positive for anti-adalimumab antibodies if the levels were greater than the threefold value (10.5 AU/ml) of the detection limit (3.5 AU/ml) on at least 1 occasion. The overall intra-assay and interassay coefficients of variation (CV) were calculated at 6.6% and 11.8%, respectively. All baseline samples taken before starting adalimumab therapy were negative for anti-adalimumab antibody. Determination of plasma trough levels of adalimumab. Plasma trough levels of adalimumab were determined using sandwich ELISA according to the manufacturer's instructions (Progenika Biopharma, SA, Derio, Spain). A human monoclonal F(ab′)2 fragment to adalimumab was engineered in this assay, in contrast to polyclonal antibodies used in other assays. In brief, 100 μl of plasma samples was added to the wells precoated with an anti-adalimumab human F(ab′)2 fragment and then incubated for 1 hour at RT. After removal of the content of each well by decanting, 200 μl of diluted wash buffer was added and later removed by decanting for a total of 3 washes. After eliminatio [file 6656121.f1.docx]

**Supplementary Materials and Methods**

***Assessments of plasma levels of antibodies against adalimumab***

Antibodies against adalimumab were detected by bridging ELISA (Progenika Biopharma SA, Derio, Spain). Briefly, 100μl of samples were added to ELISA-corresponding wells pre-coated with adalimumab, and then incubated for 1 hour at room temperature (RT, 20-25℃). After removal of the content of each well by decanting, 200μl of diluted wash buffer was added and later removed by decanting for a total of 3 washes. Subsequently, 100μl of horseradish peroxidase (HRP)-conjugated adalimumab was added to each well, and then incubated for 1 hour at RT. After 3 washes with diluted wash buffer solution, 50μl of substrate solution was added to each well, and incubated for 30 minutes at RT. Finally, 50μl of stop solution was added to each well and then thoroughly mixed. The absorbance (OD) to a 450nm wavelength in each well was read within 15 minutes. Patients were defined as positive for anti-adalimumab antibodies if the levels were greater than 3-fold value (10.5AU/ml) of the detection limit (3.5AU/ml) on at least 1 occasion. The overall intra-assay and inter-assay coefficient of variation (CV) was calculated at 6.6% and 11.8%, respectively. All baseline samples taken before starting adalimumab therapy were negative for anti-adalimumab antibody.

***Determination of plasma trough levels of adalimumab***

Plasma trough levels of adalimumab were determined using sandwich ELISA according to the manufacturer’s instructions (Progenika Biopharma SA, Derio, Spain). A human monoclonal F(ab’)_2_ fragment to adalimumab was engineered in this assay, in contrast to polyclonal antibodies used in other assays. In brief, 100μl of plasma samples were added to the wells pre-coated with an anti-adalimumab human F(ab’)_2_ fragment, and then incubated for 1 hour at RT. After removal of the content of each well by decanting, 200μl of diluted wash buffer was added and later removed by decanting for a total of 3 washes. After elimination of the diluted wash buffer solution, 100μl of HRP-labeled anti-adalimumab F(ab’)_2_ fragment was added to each well, and incubated for 1 hour at RT. After 3 washes with diluted wash buffer solution, 50μl of substrate solution was added to each well, and then incubated in the dark for 30 minutes at RT. Finally, 50μl of stop solution was added to each well, and thoroughly mixed. The absorbance (OD) to a 450nm wavelength in each well was read within 15 minutes. The minimal detectable levels were 0.024μg/ml for adalimumab. The overall intra-assay and inter-assay CV were calculated at 6.1% and 5.1% respectively.

**Supplementary Tables**

**Supplementary Table S1.** Clinical characteristics of RA patients with and without ADAb in the cohort-2 (Hospital B) ^a^

|  | **ADAb-positive (n=24)** | **ADAb-negative**  **(n=26)** | ***P*-value** |
| --- | --- | --- | --- |
| Mean age at entry of study, years | 53.4 ±10.0 | 53.7 ± 10.2 | 0.934 |
| The proportion of female (%) | 22 (91.7%) | 23 (88.5%) | 1.000 |
| Disease duration, years | 13.3 ± 5.1 | 13.0 ± 5.3 | 0.806 |
| RF positivity (%) at baseline | 18 (75.0%) | 22 (84.6%) | 0.490 |
| ACPA positivity (%) at baseline | 16 (66.7%) | 19 (73.1%) | 0.760 |
| DAS-28 at baseline | 6.63 ± 0.81 | 6.54 ± 0.81 | 0.690 |
| Daily steroid dose (mg) at baseline | 7.0 ± 1.8 | 6.6 ± 1.7 | 0.493 |
| Weekly MTX dose (mg) at baseline | 13.0 ± 2.2 | 12.6 ± 2.3 | 0.508 |
| csDMARDs at baseline |  |  |  |
| Methotrexate | 24 (100%) | 26 (100%) | － |
| Sulfasalazine | 18 (75.0%) | 21 (80.8%) | 0.738 |
| Hydroxychloroquine | 15 (62.5%) | 18 (69.2%) | 0.767 |
| Plasma ada. levels at week 24, μg/ml | 0.02 (0.02-0.42) | 5.02 (4.20-5.98) | <0.001 |
| Poor EULAR responder at week 24 | 18 (75.0%) | 2 (7.7%) | <0.001 |
| The proportion of LDA at week 24 | 2 (8.3%) | 14 (53.8%) | 0.001 |

^a^ Data are presented as mean ± standard deviation, number (percentage) or median (interquartile range)

Abbreviations: RF, rheumatoid factor; ACPA, anti-citrullinated peptide antibodies; DAS28, disease activity score for 28-joints; MTX: methotrexate; DMARDs, disease-modifying anti-rheumatic drugs; ada., adalimumab; LDA, low disease activity, which was defined as DAS28≦3.2; EULAR, European League Against Rheumatism; poor responders are those who have either ∆DAS28 (DAS28 decrement)＜0.6 or a DAS28>5.1 at week 24 of adalimumab therapy.

Mann-Whitney U test was used for between-group comparison of numerical variables.

The χ^2^ test with Yates’s continuity correction or Fisher’s exact test was used to compare binary variables

**Supplementary Table S2.** The top 20 putative biomarkers which can stratify RA patients based on ADAb status.

| **Protein**  **(at baseline)** | **Protein**  **name** | **Penetrance frequency %**  **(Non- responders)** | **Penetrance fold change (Non- responders)** | **Frequency % differential** | **AUC** | **Protein**  **(at weeks 24)** | **Protein**  **name** | **Penetrance frequency %**  **(Non- responders)** | **Penetrance fold change (Non- responders)** | **Frequency % differential** | **AUC** |
| --- | --- | --- | --- | --- | --- | --- | --- | --- | --- | --- | --- |
| TROVE2 | Ro60/SSA ribonucleoprotein | 57.143 | 3.834 | 57.143 | 0.800 | TROVE2 | Ro60/SSA ribonucleoprotein | 66.667 | 8.429 | 66.667 | 0.944 |
| PACSIN3 | Protein kinase C and casein kinase substrate in neurons protein 3 | 28.571 | 6.116 | 28.571 | 0.514 | HNRNPA2B1 | Heterogeneous nuclear ribonucleoproteins A2/B1 | 66.667 | 5.526 | 50.000 | 0.917 |
| SSB | Lupus La protein | 28.571 | 2.601 | 28.571 | 0.600 | DDX55 | ATP-dependent RNA helicase DDX55 | 50.000 | 17.409 | 50.000 | 0.806 |
| DDX55 | ATP-dependent RNA helicase DDX55 | 14.286 | 6.009 | 14.286 | 0.514 | GADD45G | Growth arrest and DNA damage-inducible protein GADD45 gamma | 50.000 | 15.558 | 50.000 | 0.667 |
| HNRNPA2B1 | Heterogeneous nuclear ribonucleoproteins A2/B1 | 14.286 | 3.726 | 14.286 | 0.543 | PACSIN3 | Protein kinase C and casein kinase substrate in neurons protein 3 | 50.000 | 12.633 | 50.000 | 0.944 |
| GADD45G | Growth arrest and DNA damage-inducible protein GADD45 gamma | 14.286 | 3.713 | 14.286 | 0.600 | BUD31 | Protein BUD31 homolog | 50.000 | 8.035 | 50.000 | 0.667 |
| PDCL3 | Phosducin-like protein 3 | 14.286 | 3.366 | 14.286 | 0.486 | ZNF207 | BUB3-interacting and GLEBS motif-containing protein ZNF207 | 50.000 | 6.356 | 50.000 | 0.694 |
| IL18 | Interleukin-18 | 14.286 | 2.628 | 14.286 | 0.371 | PDCL3 | Phosducin-like protein 3 | 50.000 | 4.753 | 50.000 | 0.917 |
| CASP7 | Caspase-7 | 14.286 | 2.577 | 14.286 | 0.400 | NOL4 | Nucleolar protein 4 | 50.000 | 3.986 | 50.000 | 0.806 |
| EFHD2 | EF-hand domain-containing protein D2 | 14.286 | 2.420 | 14.286 | 0.486 | SSB | Lupus La protein | 50.000 | 3.919 | 33.333 | 0.694 |
| TSGA10 | Testis-specific gene 10 protein | 14.286 | 2.340 | 14.286 | 0.514 | TPM3 | Tropomyosin alpha-3 chain | 50.000 | 3.625 | 33.333 | 0.778 |
| CD96 | T-cell surface protein tactile | 14.286 | 2.321 | 14.286 | 0.429 | TPM1 | Tropomyosin alpha-1 chain | 50.000 | 3.431 | 50.000 | 0.833 |
| TGFBR2 | TGF-beta receptor type-2 | 14.286 | 2.297 | 14.286 | 0.400 | ZHX2 | Zinc fingers and homeoboxes protein 2 | 50.000 | 3.207 | 50.000 | 0.833 |
| FKBP1B | Peptidyl-prolyl cis-trans isomerase FKBP1B | 14.286 | 2.287 | 14.286 | 0.314 | TSGA10 | Testis-specific gene 10 protein | 50.000 | 2.862 | 50.000 | 0.806 |
| LRRFIP2 | Leucine-rich repeat flightless-interacting protein 2 | 14.286 | 2.244 | 14.286 | 0.486 | ZMYND8 | Protein kinase C-binding protein 1 | 50.000 | 2.589 | 50.000 | 0.694 |
| SH3GL1 | Endophilin-A2 | 14.286 | 2.243 | 14.286 | 0.457 | PSIP1 | PC4 and SFRS1-interacting protein | 50.000 | 2.497 | 50.000 | 0.722 |
| AFF4 | AF4/FMR2 family member 4 | 14.286 | 2.144 | 14.286 | 0.286 | RTFDC1 | Protein RTF2 homolog | 50.000 | 2.490 | 50.000 | 0.694 |
| CEP55 | Centrosomal protein of 55 kDa | 14.286 | 2.136 | 14.286 | 0.286 | ZNF593 | Zinc finger protein 593 | 50.000 | 2.425 | 50.000 | 0.667 |
| PHLDA1 | Pleckstrin homology-like domain family A member 1 | 14.286 | 2.122 | 14.286 | 0.286 | NFE2 | Transcription factor NF-E2 45 kDa subunit | 50.000 | 2.365 | 50.000 | 0.639 |
| TPM1 | Tropomyosin alpha-1 chain | 14.286 | 2.118 | 14.286 | 0.286 | MAGEC2 | Melanoma-associated antigen C2 | 50.000 | 2.337 | 50.000 | 0.694 |

ADAb: anti-drug antibody; AUC: the area under receiver-operating characteristic curve

**Supplementary Table S3.** A multiple logistic regression was calculated to predict the presence of ADAb based on the 20 biomarkers.


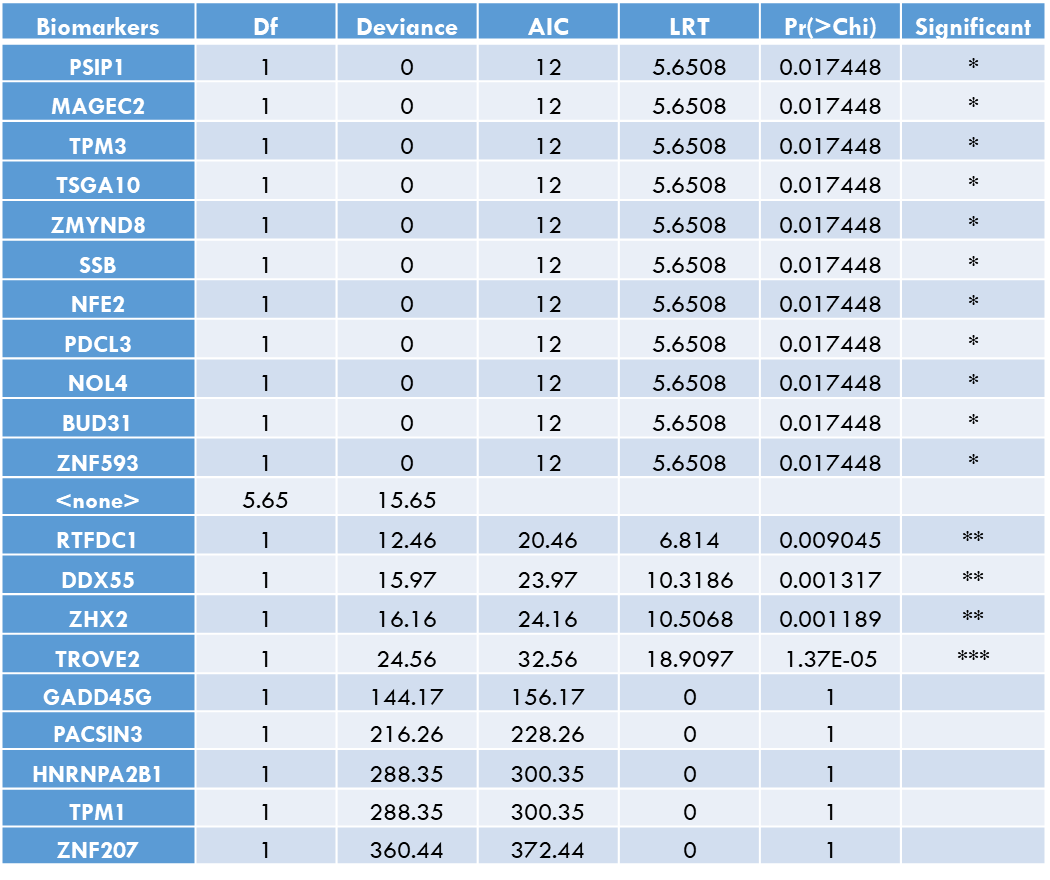


Significant codes: ‘***’ 0.001 ‘**’ 0.01 ‘*’ 0.05

**Supplementary Table S4.** The panels of biomarkers in stratifying anti-drug antibody (ADAb)-positive and ADAb-negative RA patients

| **Panel** | **Variables** | **ROC** | **Sensitivity** | **Specificity** | **Panel identified** |
| --- | --- | --- | --- | --- | --- |
| 1 | 16 | 0.651 | 0.743 | 0.657 | TROVE2, SSB, NDE1, PPP2R1B, TRIB2, PRM2, APPL1, UCHL3, GRK6, PRSS50, HOXA1, ZHX2, GADD45G, ARRB1, CXCR2, TRIM32 |
| 2 | 8 | 0.704 | 0.710 | 0.652 | TROVE2, SSB, ZHX2, PPP2R1B, PANK3, SLCO6A1, CARD9, HOMER2 |
| 3 | 8 | 0.696 | 0.710 | 0.614 | TROVE2, SSB, NDE1, ZHX2, SH3GL1, CARD9, PTPN20, KLHL12 |
| 4 | 4 | 0.725 | 0.681 | 0.710 | TROVE2, SSB, NDE1, ZHX2 |
| 5 | 4 | 0.719 | 0.681 | 0.619 | TROVE2, SSB, SH3GL1, GNAO1 |
| 6 | 16 | 0.673 | 0.681 | 0.657 | TROVE2, SSB, NDE1, ZHX2, 7GADD45G, CEP55, IDI1, LIN28A, STK32C, TRIB2, GEM, TAF6L, HOMER2, PPP2R1B, BRSK1, TWF1 |
| 7 | 4 | 0.666 | 0.681 | 0.614 | TROVE2, NDE1, SH3GL1, SSB |

Abbreviations: RA: rheumatoid arthritis; ROC: receiver-operating characteristic

**Online Supplementary Figures**

**
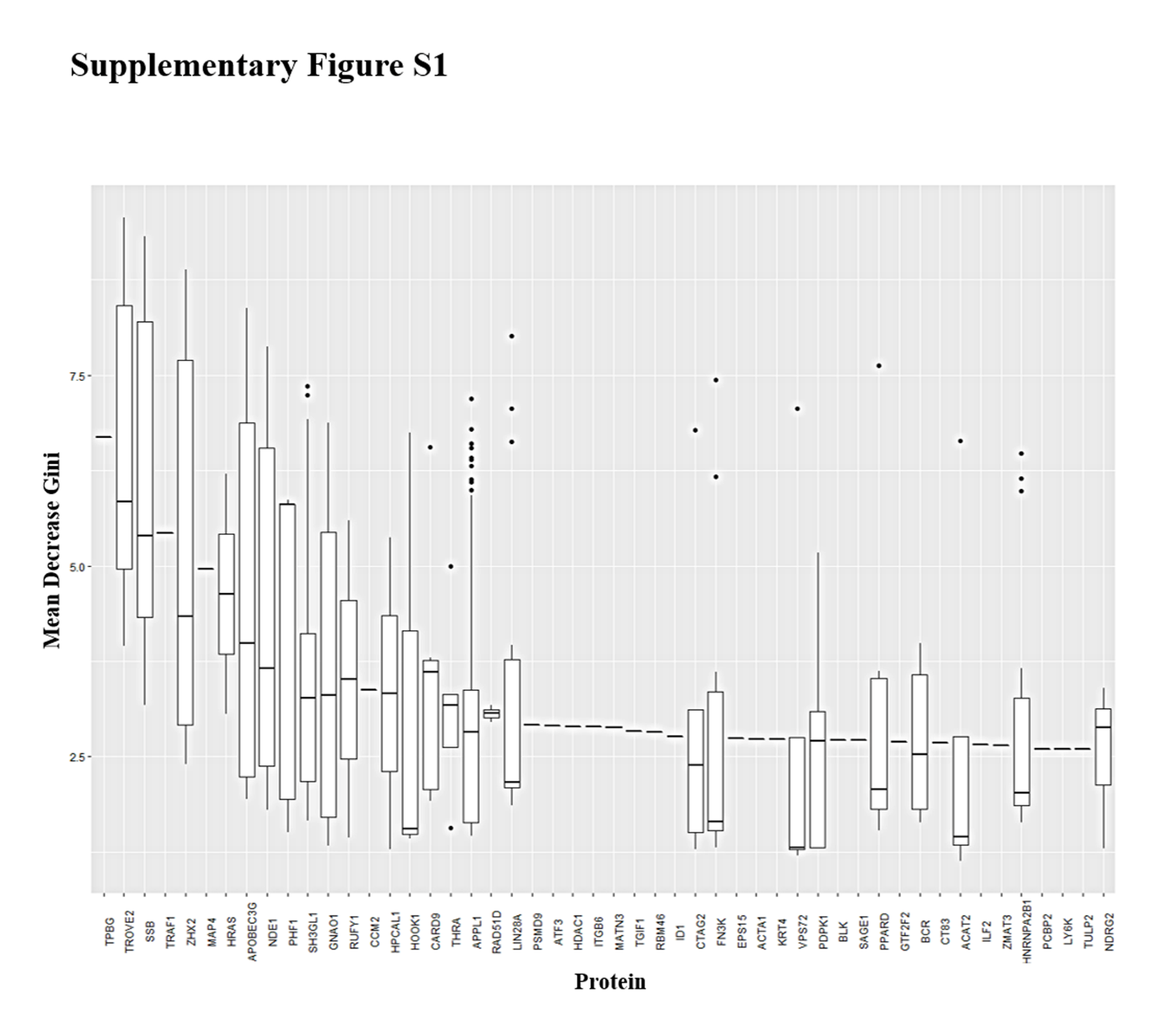
**

**Supplementary Figure S1**. The ranking of the discriminative power for anti-drug antibody (ADAb) status among the individual biomarkers based on mean decrease Gini value.


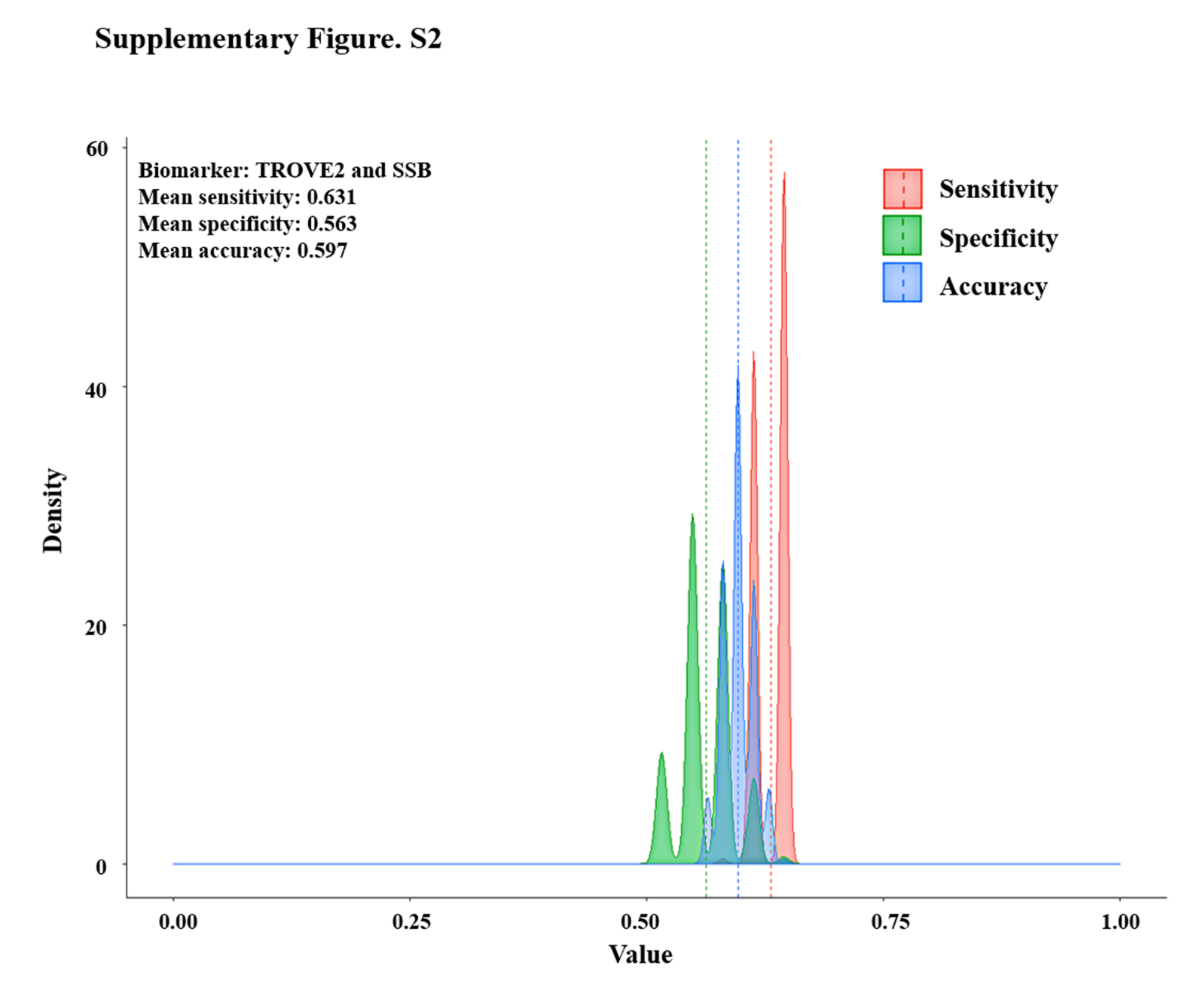


**Supplementary Figure S2**. Mean distribution plot and the stability of a panel of both TROVE2 and SSB

**
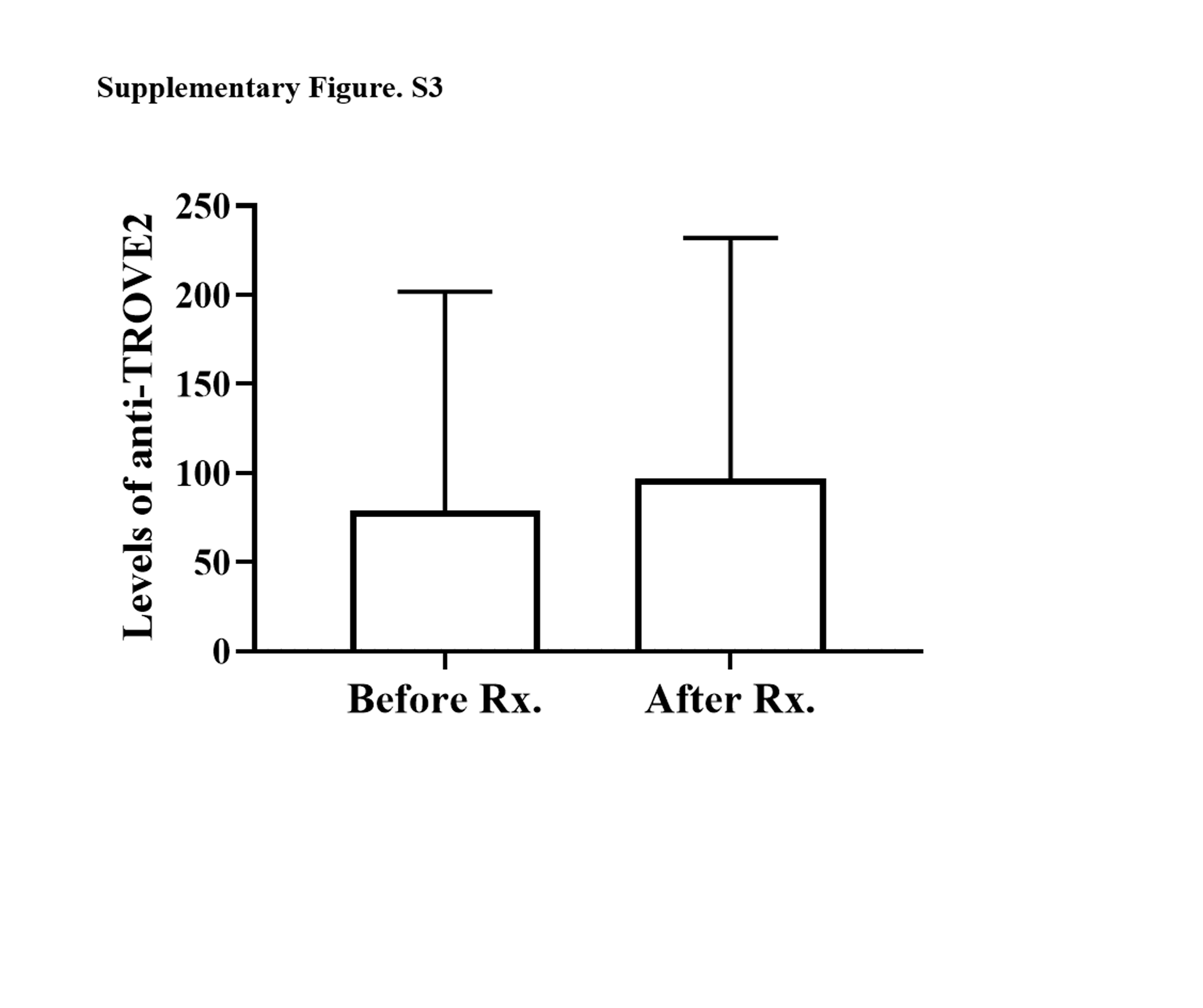
**

**Supplementary Figure. 3. Change in plasma anti-TROVE2 levels after 6-month adalimumab therapy.**

The change in anti-TROVE2 levels determined by fluorescence immunoassay before and after 6-month therapy was analyzed, and no statistical significance was found. Rx.: treatment. Bars and error bars indicate mean value and standard deviation respectively.
